# Extracellular Vesicles and Epidermal Growth Factor Receptor Activation: Interplay of Drivers in Cancer Progression

**DOI:** 10.3390/cancers15112970

**Published:** 2023-05-30

**Authors:** Enea Ferlizza, Donatella Romaniello, Francesco Borrelli, Federica Pagano, Cinzia Girone, Valerio Gelfo, Rikke Sofie Kuhre, Alessandra Morselli, Martina Mazzeschi, Michela Sgarzi, Daria Maria Filippini, Gabriele D’Uva, Mattia Lauriola

**Affiliations:** 1 Department of Medical and Surgical Sciences (DIMEC), University of Bologna, 40138 Bologna, Italy; enea.ferlizza2@unibo.it (E.F.); donatella.romaniello@unibo.it (D.R.); francesco.borrelli3@unibo.it (F.B.); federica.pagano3@unibo.it (F.P.); cinzia.girone2@unibo.it (C.G.); valerio.gelfo2@unibo.it (V.G.); rikkesofie.kuhre@studio.unibo.it (R.S.K.); alessandra.morselli4@unibo.it (A.M.); martina.mazzeschi2@unibo.it (M.M.); michela.sgarzi2@unibo.it (M.S.); dariamaria.filippin2@unibo.it (D.M.F.); gabrielematteo.duva2@unibo.it (G.D.); 2 IRCCS Azienda Ospedaliero-Universitaria di Bologna, 40138 Bologna, Italy

**Keywords:** microvesicles, exosomes, NSCLC, tumour progression, tumour microenvironment, metastasis, therapy resistance, biomarker, liquid biopsy

## Abstract

**Simple Summary:**

Extracellular vesicles (EVs), consisting of microvesicles and exosomes, serve as messengers for intercellular communication by transporting proteins and nucleic acids. In solid cancers of epithelial origin, Epidermal Growth Factor Receptor (EGFR) plays a pivotal role as a driver. In vitro studies conducted on EGFR-dependent solid tumours revealed the significant correlation between EGFR and EVs production, leading to the dissemination of EGFR itself and related molecules along with inducing cell proliferation, modifying the tumour microenvironment, facilitating metastases, and conferring resistance to treatments. Recently, liquid biopsy approaches started to exploit the interplay of EGFR and EVs in delivering proteins, RNAs, and DNAs via blood/plasma of EGFR-dependent tumour patients to evaluate their possible roles and applications as candidate biomarkers in diagnosing and monitoring tumour progression and therapy efficacy.

**Abstract:**

Extracellular vesicles (EVs) are of great interest to study the cellular mechanisms of cancer development and to diagnose and monitor cancer progression. EVs are a highly heterogeneous population of cell derived particles, which include microvesicles (MVs) and exosomes (EXOs). EVs deliver intercellular messages transferring proteins, lipids, nucleic acids, and metabolites with implications for tumour progression, invasiveness, and metastasis. Epidermal Growth Factor Receptor (EGFR) is a major driver of cancer. Tumour cells with activated EGFR could produce EVs disseminating EGFR itself or its ligands. This review provides an overview of EVs (mainly EXOs and MVs) and their cargo, with a subsequent focus on their production and effects related to EGFR activation. In particular, in vitro studies performed in EGFR-dependent solid tumours and/or cell cultures will be explored, thus shedding light on the interplay between EGFR and EVs production in promoting cancer progression, metastases, and resistance to therapies. Finally, an overview of liquid biopsy approaches involving EGFR and EVs in the blood/plasma of EGFR-dependent tumour patients will also be discussed to evaluate their possible application as candidate biomarkers.

## 1. Introduction

Extracellular vesicles (EVs) are a highly heterogeneous population of cellular-derived phospholipid bilayer membrane entities. These particles can be isolated from various human biofluids [1,2,3] and deliver complex messages by transferring cytosolic proteins, lipids, RNA, DNA, and metabolites inside and on their surface [4,5,6,7,8].

Since the first hypothesis in 1946 by Chargaff and West [9], EVs were described decades later from platelets in 1967 [10] and then observed as matrix vesicles [11]. Other independent studies reported the release of membrane vesicles from rectal adenoma microvillus cells [12], virus-like particles in human cell cultures and bovine serum [13,14] and seminal plasma [15]. In the early 1980s, tumour-originating fragments were also described, reporting the fusion of multivesicular bodies (MVBs) with the plasma membrane, leading to the release of vesicles, later named exosomes (EXOs) [16,17,18]. Finally, in 1996, Raposo et al. documented that EVs could stimulate adaptive immune responses [19].

A milestone in EVs research was the discovery of cargo RNA encapsulated within EVs, suggesting a function as a means for intercellular transfer of genetically encoded messages [20,21,22] and, more recently, cargo DNA and the EV-DNA-protein complex [23,24,25]. EVs were then isolated from most cells and body fluids, and the research field is constantly growing.

The EV population comprises different subtypes and is currently categorized based on their biogenesis and size into two main groups: large/medium EVs and small EVs (sEVs). In general, large/medium EVs include apoptotic bodies and microvesicles (MVs), while sEVs include EXOs [3,4,6,26]. The list of EV subtypes is constantly growing, including exospheres, migrasomes, small EV clusters, and the recently discovered exomeres, supermeres, and chromatimeres [27,28,29,30]. In addition to these classes, some cancer-specific subtypes of EVs have been identified: oncosomes (~100–400 nm) produced by non-transformed cells, whose contents can determine oncogenic effects, and large oncosomes (~1–10 µm) derived from malignant cells [5].

In oncology, EVs were reported as essential for tumour initiation, progression, and metastasis. Malignant cells were shown to transfer bioactive molecules to the neighbouring stroma, interfering with signalling and regulation of gene expression in the recipient cell and creating a favourable tumour microenvironment (TME) for cancer progression and metastatic spread [31,32,33,34]. EVs also mediate epithelial-to-mesenchymal transition (EMT), invasion, angiogenesis, immune modulation, and migration, and communicate over long distances, influencing normal distant cells and establishing a pre-metastatic niche [7,35,36]. EVs’ roles in metastatic growth and the removal of chemotherapeutics from cancer cells have also been extensively studied [32,37,38].

In this review, we will primarily focus on EXOs and MVs. First, we will provide an overview of EVs, their biogenesis mechanisms, and the differences in light of EGFR. Next, we will examine and analyse the interplay between EVs and EGFR. We will explore two main perspectives: namely, the molecular mechanisms of EV release consequent to EGFR activation, and, conversely, EGFR activation in relation to EV release and capture. Finally, the use of EVs as diagnostic tools and putative biomarkers in EGFR-dependent tumours will be described.

## 2. EGFR and EV Biogenesis

EGFR (or ERBB1/HER1) is a prototype transmembrane tyrosine kinase receptor activated by several growth factors. Upon ligand binding, conformational changes induce the monomeric “activator kinase” to form homo- or heterodimers with other family members (HER2, HER3, and HER4) [39]. Subsequently, the “receiver kinase” trans-phosphorylates specific tyrosine residues on the activator C-lobe, which, in turn, stimulates a cascade of effector proteins. Some of them culminate with the activation of several intracellular pathways like the Ras/Raf/Mitogen-activated protein kinases (MAPK) pathway, the phosphatidylinositol 3-kinase (PI3K)/AKT8 virus oncogene cellular homolog (AKT)/Mammalian target of rapamycin (mTOR) pathway, and the signal transducer and activator of transcription (STAT) [40]. All of them influence specific and important cellular functions: proliferation, survival, angiogenesis, inflammation, and metabolism [41]. On the contrary, for fine regulation of this receptor, other effectors trigger the negative route through CBL and E3 ubiquitin ligase for EGFR sorting and degradation in the lysosome. Moreover, recent findings have attributed structural function not only to EGFR monomers and dimers, but also to other alternative oligomers both in the basal and ligand-bound states, suggesting the complexity of EGFR signalling [42,43]. Ubiquitously expressed in different tissue types, EGFR is also one of the receptors found to be altered in several cancers, particularly breast, non-small cell lung cancer (NSCLC), glioblastoma (GBM), head and neck squamous cell carcinoma (HNSCC), ovarian, and melanoma [44,45,46,47,48,49]. EGFR signalling can also induce the loading of phosphorylated EGFR as cargo in sEVs, as well as the release of EVs [50,51].

The MVs group comprises vesicles of different sizes (~150 nm–1 µm) that directly arise from the outward budding of the plasma membrane and are involved in intercellular communication, signalling pathway activation, and cell invasion by cell-independent matrix proteolysis [4,26]. Tumour-derived microvesicles (TMVs) and oncosomes originating from cancer cells are also classified as MVs and are able to transfer bioactive molecules (namely nucleic acids, lipids, and proteins) to recipient cells, promoting cancer progression, drug resistance, and providing diagnostic markers [33,52]. MVs, being derived from the plasma membrane, carry a portion of their parent cell’s membrane content when released into the extracellular space. This allows them to reflect the cellular composition and characteristics of their origin. A combination of factors induces the formation of MVs, such as the redistribution of phospholipids and the contraction of the actin-myosin machinery [53]. ADP-ribosylation factor 6 (ARF6) leads to the activation of phospholipase D, which recruits the extracellular signal-regulated kinase (ERK) to the plasma membrane. The subsequent phosphorylation and activation of the myosin light chain kinase finally trigger the release of MVs [32,33,35,36,54]. Within this mechanism, EGFR is known to interact with ARF6 and phospholipase D2 through MAPK, also inducing MV shedding [55,56]. As a consequence, ARF6 was shown to be involved in EGFR-dependent tumours inducing invasion and EMT and linked to prognosis, further suggesting that a deregulation of MV biogenesis may contribute to cancer progression [57,58,59].

EXOs (~40–150 nm) are intraluminal vesicles (ILVs) contained in MVBs, which are released into the extracellular environment upon fusion with the plasma membrane [5,60,61]. Unlike MVs and despite sharing common intracellular mechanisms and sorting machinery, EXOs are formed through a distinct and complex intracellular regulatory process that determines their composition and functions [8,61].

**Figure 1:**
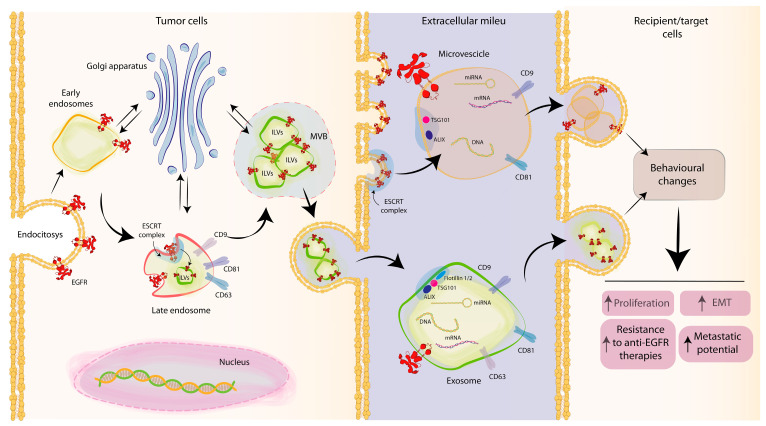
Biogenesis of microvesicles (MVs) and exosomes (EXOs) from tumour cells and effects of EV-EGFR on recipient cells. MVs directly arise from the outward budding of the plasma membrane. EXO biogenesis involves invagination of the plasma membrane, internalizing EGFR. The early endosome interacts with the Golgi apparatus and involves complex machinery (endosomal sorting complex required for transport, ESCRT, programmed cell death 6-interacting protein, ALIX, tumour susceptibility gene 101,TSG101) and different tetraspanins (CD9, CD63, and CD81), leading to a second inward invagination and the formation of the intraluminal vesicles (ILVs) contained in multi-vesicular bodies (MVBs). EXOs are then released into the extracellular milieu upon fusion with the plasma membrane.

The best-described mechanism of EXO formation is driven by the endosomal sorting complex required for transport (ESCRT), which is composed of four complexes (ESCRT-0, -I, -II, and -III) and approximately thirty associated proteins. The membrane is reorganized, becoming highly enriched in tetraspanins, mainly CD9 and CD63, while ESCRT 0 recognizes ubiquitinated proteins on the outside [61,62]. The presence of different stimuli, such as phosphatidylinositol 3-phosphate (PIP3), hepatocyte growth factor-regulated tyrosine kinase substrate, ubiquitination of endocytosed proteins, and/or the curved membrane topology, induce ESCRT I and II to initiate and drive the intraluminal membrane budding [63,64]. ESCRT III completes the process of ILV formation through programmed cell death 6-interacting protein (ALIX) and tumour susceptibility gene 101 (TSG101), which by interacting with proteins and cellular components, create a physical force that causes the vesicle scission [8,26,52,65]. Then, EXOs are released by the fusion of the MVB with the plasma membrane. RAB GTPases, including RAB11 and RAB35, or RAB27A and RAB27B, as well as SNARE proteins, e.g., vesicle-associated membrane protein 7 (VAMP7), have been implicated in the membrane fusion [26,27]. Figure 1 summarizes the biogenesis of MVs and EXOs.

In the case of EGFR, ubiquitination and phosphorylation are key factors for the sorting of the endocytosed receptor, driving it to lysosomal degradation or recycling back to the cell surface [66]. Low levels of phosphorylation drive EGFR to clathrin-mediated endocytosis and recycling, while at high levels, EGFR is ubiquitinated and recognized by the ESCRT complex, which drives it into ILVs and possibly to lysosomes for degradation. However, EGFR mutations can lead to increased auto-phosphorylation and MAPK signalling in some cancers (e.g., NSCLC), displaying abnormal ubiquitination and an increased rate of internalization and recycling [67,68].

EXOs may also form without the ESCRT complex machinery pathway, mainly involving ceramide subdomain-mediated curvature and tetraspanin family members (CD9, CD63, and CD81). Syntenin 1 has also been shown to be involved in the packaging of cargo into ILVs, as has ARF6, which can aid ILV budding, sorting of molecules, and driving MVBs and ILVs to lysosomes for degradation [35,69]. The presence of specific surface proteins in MVBs and ILVs, including EGFR, along with GTPase RAS-related protein RAB7A, HSP70−HSP90 organizing protein complexes, and members of the SNARE complex, including VAMP7 and syntaxin 7 and 8, results in the degradation of ILV contents, including EGFR itself [36,54]. On the other hand, RABs, actin, and SNARE proteins also mediate the fusion of MVBs with the cell membrane, and the subsequent release of EXOs [31,36,54,70]. Interestingly, the role of ARF6 was shown to be specifically involved in sorting and trafficking of EGFR toward degradation [71], while in aggressiveness in brain tumour-derived sEVs, Annexin1 is involved in inward budding EGFR-associated MVB [72].

## 3. EVs and EGFR Content as a Biomarker

The contents of vesicles, including MVs and EXOs, can vary depending on several factors, such as the cell type, biogenesis pathway, and physiological or pathological conditions. In general, all EVs are loaded with proteins, lipids, and nucleic acids. The different types of cargo can also be specific per vesicle and cell type, and extensive research has been carried out to characterize the content of EVs. This resulted in the assembly of several datasets from EVs studies, and nowadays, two databases are publicly available: Vesiclepedia (www.microvesicles.org (accessed on 22 March 2023)) [73] and ExoCarta (www.exocarta.org (accessed on 22 March 2023)) [3,65,74].

The methods used for isolation and characterization of EVs may yield different outcomes [6,54,75,76,77]. Ultracentrifugation, polymeric precipitation, filtration, immunoaffinity isolation, and microfluidics techniques are the most commonly used methods that are not necessarily mutually exclusive, and combinations are recommended [76,78,79]. For example, sedimentation by ultracentrifugation procedures, depending on the density or ‘‘cargo’’, may lead to the presence of some small EVs in the large vesicle pellet, while some larger particles may remain in the upper part of the tube [80]. Differently, filter-based methods with low-molecular-weight filters (0.2 µm), employed to enrich for the smaller EV fraction, may lead to deformation or breakup of larger vesicles or platelets, thereby potentially skewing results [78].

This situation is further complicated by EV heterogeneity. The current purification methods may be unable to identify EVs based on their subtype or biogenesis, due to overlapping sizes and a lack of well-established genetic markers [4,26]. Furthermore, different subpopulations of MVBs coexist simultaneously in cells. For example, in HeLa cells two distinct EV populations have been reported after stimulation with EGF [81]: a population of CD63-positive endosomes containing EGFR and another subset of MVBs still positive for CD63 but negative for EGFR cargo.

Despite the difficulties in standardization, mandatory for a possible clinical application, EVs are considered a good source for tumour molecular profiling compared with cell-free nucleic acids because EVs protect nucleic acids from degradation [5,82]. Moreover, tumour cells release EVs with an altered cargo profile, influencing intercellular communication during metastatic spreading [31,83]. This is also reflected by qualitative and quantitative changes in sEV populations, which were identified in the blood of cancer patients, corroborating a function of EVs as diagnostic and prognostic markers [52,78,84,85,86,87,88]. In line with this, increased MV levels have been detected in the plasma of patients suffering from gastric, lung, breast, pancreatic, colorectal, and prostate cancers, as well as in hematologic malignancies [33,89,90,91,92,93,94].

Hereafter, the review will focus on EXO cargo, namely, proteins, RNAs, and DNA found within EXOs and MVs, with a focus on EGFR-dependent tumours. EV-EGFR as a biomarker in body fluids will also be discussed.

### 3.1. Proteins

Proteomics approaches in primary cell cultures or biofluids yielded catalogues of proteins in different types of EVs. Notably, the proteomic profiles obtained were significantly influenced by the isolation methods used for EVs, resulting in variable homogeneity of EVs and subfractions [79]. Furthermore, the same cell type may secrete different subgroups of vesicles depending on environmental factors (e.g., oxygen tension) or an activating stimulus [3]. Despite the partial overlapping results, some proteins can be considered pan-EV markers (i.e., common for most EVs), while other proteins and post-translational modifications are specific for vesicle localization, cellular origin, and mechanism of secretion. As expected, either cytoskeletal proteins and plasma membrane proteins or proteins involved in vesicle trafficking are highly abundant in EVs and are currently used as markers of EV subpopulations (Table 1).

In addition to the various proteins involved in biogenesis (e.g., ESCRT complex, ALIX, TSG101), sorting (e.g., glycan binding, tetraspanins) [3,26,65,96,97,98], and fusion with membrane (GTPase RAS-related protein, SNARE complex, ARF6, VAMP7, and syntaxin 7 and 8) [52,69] usually used to characterize EV populations, EVs contain specific stress proteins (heat shock proteins; HSP70−HSP90), antigen presentation complex (MHC I and MHC II) and transmembrane proteins (lysosomal-associated membrane protein, LAMP1; transferrin receptor, TfR), including the membrane receptor EGFR.

Li et al. [99] analysed EVs secreted by nasopharyngeal carcinoma (NPC) cells and detected an EXO subpopulation enriched for membrane CD9 and CD63 and non-membrane ALIX and TSG101 proteins. Subsequently, the authors evaluated the role of EGFR-rich EVs both in vivo (tumour tissues) and in vitro (high and low metastatic NPC cell lines). Additionally, they found that EGFR was overexpressed in metastatic NPC cell lines and patients. Furthermore, EGFR-rich EVs secreted by metastatic NPC cell lines induced cellular up-regulation of EGFR and abnormal signalling activation, which, in turn, are responsible for EMT and shorter survival in xenografted mice treated with secreted EGFR-rich EVs [99]. The importance of EXO-EGFR and related proteins was also reported in lung cancer and EMT. Jouda et al. [100], showed that EXO-EGFR from EGFR mutated NSCLC patients can induce the activation of the PI3K/AKT/ mTOR pathway in A549 cells. The authors also demonstrated an increase in vimentin, nuclear factor erythroid 2-related factor 2, and P-cadherin, suggesting that EXOs may act as mediators of EMT and tumour invasion.

The oncogenic role of EGFR in triggering the release of EXO-like extracellular vesicles and the evaluation of phosphoprotein content both in vivo and in vitro was also reported [101]. Both wild-type and mutant EGFR were detected in EVs isolated in the plasma of patients affected by GBM and in GBM xenograft mice, reflecting the mutations found in the tumour. After highlighting the ability of GBM-EVs to induce the release of EGFR and phospho-EGFR, the authors also reported similar findings in additional cancer cell lines, including A431 (epidermal), MDA-MB-231 (breast), BxPC3, PANC-1 (pancreatic), PC-3 (prostate), HCT116, DLD-1, and CaCo-2 (colon). In A431 cells, TGFα treatment boosted phosphorylation of both membrane EGFR and EV-engulfed EGFR. On the other hand, panEGFR inhibitors reduced phosphorylation only on the membrane located EGFR, but intriguingly increased the pEGFR activation recovered as cargo in the EV. Remarkably, the inhibition of both the EXO biogenesis and the apoptotic caspase cascade reduced the EVs release, suggesting a possible shared mechanism between EXO biogenesis and apoptosis. Thus, in EGFR-dependent cancer cells, blocking the oncogenic signals may lead to cellular stress that stimulates both exosomal and apoptotic pathways [101].

### 3.2. RNA

A milestone in EV research was the discovery of cargo RNA, suggesting its function as a means for intercellular transfer of genetically encoded messages.

The RNAs initially discovered and most studied in EVs are messenger RNA (mRNA) and microRNA (miRNA) [5,6,102]. The role of EV-RNAs is also emerging in liquid biopsy, as a promising non-invasive source for molecular testing, complementing and improving the use of circulating tumour cells and cell-free DNA (cfDNA) analysis [33,102]. In addition, enrichment analysis observed that several miRNAs were almost undetectable in colorectal cancer cell lysates but were found at high levels in the respective cell-derived MV and EXO [103]. Usually, the absence or minor amounts of ribosomal 18S and 28S in EVs are reported. However, with the rapid advances in deep sequencing or Next Generation Sequencing (NGS) techniques, fragments of rRNA, as well as long and short non-coding RNA and tRNA fragments, have been found in EVs [1,65]. Currently, the Vesiclepedia database includes over 27,000 and 10,000 entries mRNAs and miRNAs, respectively [73]. The general consensus is that EVs protect RNA from digestion in the extracellular environment. Furthermore, different ribonucleoproteins, as well as high- and low-density lipoproteins, can stably associate with RNA species [3,65,82].

In oncology, EV-RNAs are studied both as potential biomarkers in blood/plasma/serum or as emergent treatment strategies for malignant neoplasms. Table 2 reports RNAs studied in cell cultures or as biomarkers. For example, EV-miRNA Let-7b-3p, miR-150-3p, miR-145-3p, and miR-139-3p were suggested as biomarkers in the plasma of colon, breast, or lung cancer patients [104,105]. In line with this, Ogata-Kawata et al. [106] revealed that miR-23a, miR-1246, and miR-21 differ between EVs from CRC patients and controls. Furthermore, miR-486 was significantly enriched in serum EV-miRNA of CRC patients and associated with shorter survival and liver metastases [107,108,109]. Furthermore, Cha et al. [110] evaluated eight mRNA markers (*MYC*, *VEGF*, *CDX2*, *CD133*, *CEA*, *CK19*, *EpCAM*, and *CD24*) extracted from plasma EVs, showing statistically significant differences between healthy subjects and CRC patients for the combination of *VEGF* and *CD133*. Differently, Krug et al. [111] demonstrated that the measure of circulating tumour DNA (ct-DNA) and EXO-RNA was more sensitive than ct-DNA alone to detect EGFR mutations in advanced NSCLC. Nevertheless, whether enough RNA molecules are shuttled to EV to functionally influence the recipient cell remains to be proven.

In NSCLC, Xia et al. [112] suggested the possible use of EXO-miRNA as markers to identify patients with wild-type or mutated EGFR. In particular, the authors highlighted 96 EXO-miRNAs differentially expressed between the serum of healthy and NSCLC patients. Interestingly, 40 of them were also different between patients with mutated EGFR, including miR-142-5p, -592, -217, -451b, and -150, already reported as putative biomarkers. Moreover, the authors searched for EXO-miR-260 and -miR-1169 in cancer cell lines showing the same trend and expression as in the serum of patients, suggesting a correlation between downregulation of EXO-miR-260 and mutated EGFR, while upregulation of EXO-mir-1169 may identify patients with wild-type EGFR. Finally, EVs were also reported to mediate the transfer of long noncoding RNAs implicated in the pathological process of lung pulmonary fibrosis [113], which correlated with the presence of missense polymorphisms in members of the EGFR family [114].

**Table 2:** Some of the RNAs found in MVs and EXOs and studied in cell culture or as liquid biopsies biomarkers.

Target/Cargo	RNA Type	EVs	Cancer Type	Cell Line/Tissue	References
let-7-b3p, miR-150-3p, miR-145-3p, miR-139-3p	miRNA	sEV	Colon/Breast/Lung	Plasma	[105]
miR-486, miR-548c	miRNA	EXO	Colorectal	Serum	[107,108,109]
miR-1246, miR-21, miR-23a	miRNA	EXO	Colorectal	Serum/plasma	[106]
miR-92a	miRNA	EXO	Colorectal	Serum	[115]
*MYC, VEGF, CDX2, CD133, CEA, CK19, EpCAM, CD24*	mRNA	EXO/MV	Colorectal	Plasma	[110]
miR-1246, -23a, -200c, -203a, -19a, 7641	miRNA	EXO/MV	Colorectal	LM1863	[116]
BCAR4	lncRNA	EXO	Colorectal	Serum	[117]
*KRTAP5-4, MAGEA3*	mRNA	EXO	Colorectal	Serum	[117]
miR-142-5p, -592, -217, -451b, -150, -260, -1169	miRNA	EXO	NSCLC	Serum	[112]
linc00152	lncRNA	EXO	Gastric cancer	Plasma	[118]
let-7a	miRNA	EXO	Breast	HCC70	[119]
miR-145	miRNA	EXO	Breast	T-47D	[120]
miR-214	miRNA	EXO	Gastric	SGC7901	[121]
KRAS^G12D^ (target)	siRNA	EXO	Pancreas	PANC-1, BxPC-3, Capan-1, MIA PaCa-2	[122]
TPD52 (target)	siRNA	EXO	Breast	SKBR3	[123]
miR-494-3p	miRNA	EXO	NSCLC	Plasma/NCI-H1975, HCC827	[124]
miR-6087, miR-99b-5p, miR-7641, miR-378a-3p, miR-25-5p, miR-1293, miR-184, miR-3913-5p	miRNA	EXO	NSCLC	Plasma/H1975	[125]
miR-26a/b	miRNA	EXO	Gastric	SGC7901	[126]

m, messenger; mi, micro; EXO, exosome; MV, microvesicle; NSCLC, non-small cell lung cancer; sEV, small extracellular vesicle.

### 3.3. DNA

Despite most of the EV literature focusing on proteins and RNAs, numerous studies have recently reported the presence of DNA either associated with the surface of EVs or within their lumen [4]. Table 3 reports DNA genes studied in EVs. In 2013, one of the first studies reported the presence of genomic and mitochondrial DNA in human plasma EVs, and in 2014, mutant KRAS and TP53 DNAs were found in serum EXOs collected from cancer patients [24,127]. Moreover, recent findings have shown that horizontal transfer of mitochondrial DNA can occur through EVs. In detail, mitochondrial DNA carried within EVs has been found to promote the reactivation of dormant cancer stem-like cells, resulting in resistance to endocrine therapy [128].

Traditionally, it was believed that EV-DNA was the passive result of cell death by apoptosis or necrosis. However, recent studies have suggested an interesting hypothesis about the capability of EVs to also store and remove damaged DNA, potentially contributing to the maintenance of DNA integrity and homeostasis [129]. Additionally, Montermini et al. reported the EXO-DNA release as a response to cellular stress [101]. However, the biological role of EV-DNA requires further investigation.

**Table 3:** DNA found in MVs and EXOs.

DNA	EV	Cancer/Normal	Cell Line/Tissue	Reference
BCR/ABL	EV	Chronic Myelogenous Leukaemia/Healthy	Plasma/VSMC, HEK293, K562	[23]
KRAS, TP53	EXO	Pancreatic cancer	Serum/Panc-1, T3M-4	[24]
APC, KRAS, TP53, PIK3CA	MV, EXO	Colorectal cancer	Tissue biopsy	[130]
TGFBR2	EXO	Colorectal cancer	HCT116	[131]
HRAS	EXO-like	intestinal epithelial cells and fibroblasts	IEC-18, RAS-3, RAT-1	[132]
EGFR	EXO-like	epidermoid cancer	A431	[101]
EGFR	EXO	NSCLC	Plasma and tissue biopsy	[111]
EGFR T790M	EXO	NSCLC	Plasma	[133]
KRAS	EXO	Pancreatic cancer	Plasma	[134]
KRAS^G12D^, TP53^R273H^	EXO	Pancreatic cancer	Plasma	[135]
BRAF^V600E^	EV	Melanoma	Plasma	[136]
TP53, MLH1, PTEN	MV, EXO	Prostate cancer	Plasma/LNCaP, PC-3, RC92a/hTERT	[137]
MYC	L and S-EV	Prostate cancer	Plasma/PC3, U87, U2OS-H2B-GFP	[138]
DROSHA, LIG4, MACROD2, SATB1, RASSF6, BIRC2	EXO	Ovarian cancer	Plasma/OVCAR-8, OVCAR-5, FTE	[139]

APC, Adenomatous Polyposis Coli; BCR/ABL, break-point cluster region v-abl Abelson murine leukaemia viral oncogene; BIRC2; baculoviral IAP repeat containing 2, BRAF, B-Raf proto-oncogene serine/threonine kinase; DROSHA, drosha ribonuclease III; EXO, exosome; HRAS, HRas proto-oncogene, GTPase; KRAS, Kirsten rat sarcoma virus; l-EV, large extracellular vesicle; LIG4, DNA ligase 4; MACROD2, mono-ADP ribosylhydrolase 2; MLH1, mutL homolog 1; MV, microvesicle; PIK3CA, phosphatidylinositol-4,5-bisphosphate 3-kinase catalytic subunit alpha; PTEN, phosphatase and tensin homolog; RASSF6, Ras association domain family member 6; SATB1, SATB homeobox 1; s-EV, small extracellular vesicle; TGFBR2, transforming growth factor beta receptor 2; TP53, tumour protein p53; VSMC, vascular smooth muscle cells.

Reports have also shown that EV-DNA secretion varies under different conditions, leading to changes in the recipient cells. Lee et al. reported that full-length oncogenic H-RAS DNA was transferred in vitro via EVs, changing the behaviour of the recipient cells [132]. By comparing the emission of rat intestinal cells (IEC-18) and h-RAS mutated cells (RAS-3), the authors reported the increased excretion of EXO-like vesicles in non-transformed fibroblast (RAT-1), allowing the uptake of the oncogenic h-RAS with a concomitant mitogenic response. Nevertheless, whether the tumour EXO-gDNA could be horizontally transmitted with the consequent changes in the recipient cells is still controversial.

EV-DNA was found in different biological fluids and cell cultures, suggesting it is a promising candidate for liquid biopsy [29,134,140,141,142]. In cancer patients, EV-DNA is being investigated in several applications, mainly as a biomarker or to monitor disease and treatment response in liquid biopsy [139,143]. Moreover, the search by NGS for common hotspot mutations (BRAF, EGFR, and KRAS) showed a greater sensitivity if compared to circulating free DNA or circulating tumour DNA. The authors further claimed that cfDNA in plasma may be mainly present in EXOs [141].

In 2017, Allenson and colleagues found a KRAS mutation in EXOs of pancreatic ductal adenocarcinoma and associated it with disease free survival [134]. Moreover, the detection rates of mutant KRAS in EXOs were higher in EXOs as compared to cfDNA. Therefore, EV-DNA is now also considered a promising liquid biopsy approach for diagnosis, prognosis, and monitoring of treatment responses, and studies have reported its use in patients affected by pancreatic, ovarian, pheochromocytoma, and colorectal cancer [133,144,145,146,147]. In line with this, recent studies have focused on the role and usefulness of EXOs-DNA in liquid biopsy to detect mutations related to EGFR [148,149]. EGFR mutations in EXOs of malignant pleural effusion of lung adenocarcinoma patients reported 100% agreement for EGFR genotyping with the primary tumour [149]. Moreover, by analysing EV-DNA, it was possible to detect three additional T790 mutant cases in patients sensitive to EGFR-TKI and two more cases in patients with acquired resistance, suggesting that liquid biopsy using EV-DNA is promising for the detection of low-rate EGFR mutations in lung cancer. The utility of EV-DNA from liquid biopsies in lung adenocarcinoma was also confirmed by Park et al. in bronchial wash [150]. The T790M mutation rate was high, confirming that bronchial wash-derived EVs can be usefully analysed to detect DNA EGFR mutations, thus implying that EV-DNA analysis may provide valuable information for the management and treatment of lung cancer.

### 3.4. EV-EGFR in Body Fluids

In cell–cell communication, EVs have been shown to reflect the composition of their host cells [151,152]. Probably due to physiological alteration, it has been estimated that the blood of cancer patients contains around 4000 trillion EXOs, twice as much as compared to normal human blood, which makes them a potential non-invasive biomarker for different cancer screenings [153,154]. Many studies are focusing mostly on miRNAs and protein content analysis of circulating EVs. For example, in lung adenocarcinoma and lung squamous cell carcinoma, researchers are correlating specific EXO miRNAs with stages and histological subtypes [155]. As a matter of fact, EXOs express a high level of miRNAs, which are known to influence mRNA stability and TME [156]. The potential diagnostic power of such molecules has been shown in the literature: miR-139-5p, miR200b-5p, miR-378a, miR-126, and miR-379 allow for discrimination between healthy and lung cancer smokers, while other specific EXO miRNAs are useful to distinguish between lung adenocarcinomas and granulomas [157]. Recently, much attention has been concentrated on the presence of EXO-EGFR at DNA, RNA, and protein levels as diagnostic, prognostic, and therapeutic markers. Indeed, early detection of the EGFR mutation profile would lead to a prompt treatment schedule. An exhaustive table on the evaluation of EV-EGFR in liquid biopsy in different tumour types is provided by Frawley at al. [7]. Using a microfluidic electrochemical immunosensor system, Ortega et al. indirectly detected the EXO EGFR level for the diagnoses of 20 healthy patients and 30 breast cancer patients [158]. With a different flow cytometry-based approach, Wang et al. compared the EV EGFR content in several sample sera to identify glioma patients in correlation with tumour grade [159]. For diagnostic purposes, the first FDA approved liquid biopsy test was the Cobas EGFR Mutation Test v2^®^ (Roche Diagnostics Inc., Basilea, Switzerland), which analyses the EGFR gene mutations in cfDNA [160]. Based on the study performed by Fernando et al., 93% of cfDNA derives from EXOs [141]. However, such a test showed only 58% sensitivity and 80% specificity. Subsequently, further studies suggested that combining the mutational profiles of exosomal RNA/DNA and cfDNA would improve both sensitivity and specificity [133].

## 4. Role of EGFR on Cargo Trafficking and Modulation of the Tumour Microenvironment

EVs are generated by the endolysosomal pathway and released by several cell types, including tumour cells. EVs resemble the phenotypic features of the cell of origin and carry different bioactive molecules, cytokines, and receptors, including the ERBB receptor family [161,162]. Zanetti-Domingues et al. reported a central role for EGFR receptor signalling in EV trafficking and its loading as cargo [35,69]. Several studies demonstrated a high abundance of circulating EXOs in the plasma of cancer patients compared to healthy ones [154,163]. While in the latter, EXO contributes to maintaining stable homeostasis, in the former, EXO release is associated with tumour progression, metastasis, and drug resistance, and is able to deliver active cargo to recipient cells in an autocrine, paracrine, or endocrine manner [31,70,151,164]. Indeed, EGFR members and their ligands are expressed in sEVs released by cancer cells [7,165]. Through a process known as “horizontal oncogene transmission”, Al-Nedawi et al. demonstrated, in 2008, the presence of a constitutively activated mutant isoform of EGFRvIII in U373 GBM cell-derived EVs, which appeared to be functional once internalized by the recipient cells, increasing MAPK, AKT, PDK1, and RAF signalling as well as VEGF expression [34]. In addition, subsequent studies showed a different proteome composition in the sEV driven by the mutant EGFR form compared with the wild-type receptor, influencing a pro-tumoral microenvironment [166].

An altered sEV proteome was also found in NSCLC, contributing to resistance to anti-EGFR therapies [167]. In vitro culture of PC9 cells, harbouring the EGFR-T790M mutation and insensitive to gefitinib treatment, released sEVs containing specific molecules of the AKT/mTOR pathway, influencing the drug sensitivity of the recipient cells but also enhancing proliferation and invasion. Moreover, sEVs secreted by gefitinib-treated PC9, can induce autophagy in autologous cells, decreasing cisplatin activity and allowing recipient cells to survive through an antagonistic interaction [168].

EXOs derived from patients with EGFR-positive lung adenocarcinoma caused, in recipient cells, vimentin overexpression, extracellular matrix degradation, and activation of PI3K/AKT/mTOR, a well-known pathway involved in the EMT process [100]. The link between EGFR-mediated EVs biogenesis and EMT was also demonstrated by Fujiwara et al. in HSC-3 oral squamous cell carcinoma; cells under EGF stimulation induced both EMT and increased EVs secretion, which, in turn, led to the expression of EMT markers in recipient cells [50,169]. The same phenomenon was seen in HNSCC under TGF-α stimulation [170]. Stromal cells and TME are also another target affected by EV release in order to sustain neoplastic features, especially where inflammation is one of the major malignant risk factors, like in colorectal cancer. EGFR was shown to be overexpressed in inflammatory bowel disease (IBD)-associated colorectal adenocarcinoma, and indeed, an increased level of EGFR was found in IBD mice-derived colorectal EVs which were also internalized by NIH3T3 fibroblast cells [171,172]. NIH3T3, in turn, demonstrated an increased protein level of EGFR after EVs incorporation associated with high ERK phosphorylation, increased proliferation, and KI67 expression [125,171]. Thus, EVs are the principal mediator of tumour–TME communication, interacting not only with fibroblasts, but also with other important recipient cells, such as endothelial or immune cells, shaping them in favour of tumour growth. The final effect is translated into pro-angiogenic and immune suppression signalling [35]. In particular, EVs influence T regulatory cells (Tregs) and myeloid-derived suppressor cells (MDSCs). For example, it has been demonstrated in NSCLC that EGFR+ sEVs induce tolerogenic dendritic cells, which, in turn, suppress T cell activation [173].

Thus, cancer cell-derived EVs, depending on their specific patterns or cargo, can reprogram long-distance and organ-specific cells to promote growth and invasion, form a pre-metastatic niche, modulate TME, and confer drug resistance (Figure 1).

## 5. EV-EGFR in Resistance to Therapy and as Vehicles of Treatment

### 5.1. Resistance to Therapy

Recently, EV-EGFR and related proteins have also been described as involved in resistance to therapies. Choi et al. [167] demonstrated significant differences in the proteome profiles of EVs released by sensitive NSCLC (PC9) compared to gefitinib-resistant cells (PC9R) due to an acquired EGFR mutation (T790M). In particular, the AKT/mTOR signalling pathway was hypothesised as a key component actively involved in gefitinib resistance. The importance of EVs in therapy resistance was also reported in breast cancer. HER2-positive EVs were shown to bind to monoclonal antibody-based drugs (trastuzumab), thereby reducing therapy bioavailability to their target tumour cells [94,174,175]. A different mechanism was shown by Martinez et al. [176], highlighting the increased levels of the immunosuppressive proteins transforming growth factor beta 1 and programmed death-ligand 1 in EVs in HER2-overexpressing drug-resistant cells and the serum of patients who do not respond to HER2-targeted drug treatments.

EXO-miRNAs were also associated with resistance to treatments against EGFR. A potential role of miR-494-3p as a marker of osimertinib resistance in NSCLC was reported by Kaźmierczak et al. [124] by analysing EXO-RNA from plasma sampled at baseline and after disease progression in patients with mutated EGFR T790M NSCLC. The expression of EXO miR-494-3p was significantly increased in progression samples compared with baseline samples. Furthermore, the authors confirmed that miR-494-3p was significantly upregulated in two EGFR-mutant NSCLC cell lines refractory to osimertinib, demonstrating a partial role of miR-494-3p in conferring resistance to treatment in vitro. Li et al. [125], analysing sensitive and resistant NSCLC cell lines, demonstrated that EXO-derived miRNAs may be involved in the mechanisms of resistance. Levels of EXO-miR-6087, miR-99b-5p, and miR-7641 were upregulated, and miR-378a-3p, miR-25-5p, and miR-1293 were downregulated in sensitive cells and linked to the RAS-MAPK signalling pathway, a member of the bypass pathways involved in drug resistance in EGFR-TKI therapy for NSCLC. Furthermore, analysing blood samples from 64 NSCLC patients with EGFR mutations (on exons 19 and 21), the authors reported that the upregulation of EXO-miR-184 and -3913-5p may indicate the development of drug resistance in patients.

Despite EGFR being early linked to oncogenesis in GBM, therapies exploiting it as a target are still not as efficient as in other types of cancer (e.g., NSCLC) [177]. Chi et al. evaluated the RNA content of tumour-derived EVs in patients unresponsive to dacomitinib [178]. The authors found 32 mRNAs differentially expressed between sensitive and non-sensitive patients, including *LAMTOR2,* an activator of MAPK and mTOR signalling, and *CSF1*, which encodes macrophage colony-stimulating factor. Tamizolide resistant GBM cells were shown to induce resistance in sensitive cells by transferring miR-1238 through EXOs [179]. miR-1238 reduced the activity of caveolin-1, a tumour suppressor gene, also through its interaction with EGFR. Regarding the role of EXOs in promoting metastasis, Zhang et al. [126] reported that EGFR delivered via EXOs may regulate TME, promoting gastric cancer liver metastasis. EGFR was present in EXOs of gastric adenocarcinoma cells and enriched in EXOs derived from gastric cancer patients serum, but not in EXOs of healthy human serum. The levels of EGFR in both cancer cells and EXOs were strongly decreased by transfection of siRNA targeting EGFR, confirming the importance of EGFR in facilitating protein transfer of EGFR itself. The authors then demonstrated that gastric cell-EXOs significantly promote HGF and EGFR expression in mixed liver cells while suppressing miR-26a/b expression, suggesting the regulation of the microenvironment of the liver to prepare favourable conditions for future metastasis.

Furthermore, EV-DNA was reported to be involved in resistance to therapy. Crow et al. isolated EXO-gDNA from ovarian cancer cells resistant to carboplatin treatment [180]. The authors further demonstrated that tumour-derived EXOs induced an EMT phenotype in sensitive cells, leading to the development of subpopulations of platinum-refractory cells.

### 5.2. Vehicles for Treatment

The use of EVs as cargo vehicles, mainly for miRNA or siRNA, has been explored as a potential treatment option for cancer. This approach involves using EVs to deliver therapeutic RNA molecules to cancer cells [181]. For example, EXOs containing the tumour suppressor miRNA let-7a were able to successfully inhibit EGFR-expressing cancer cells in vitro and decrease tumour growth in xenograft breast cancer mice in vivo [119]. Interestingly, the authors modified EXOs with GE11 peptide, which binds membrane EGFR more efficiently than EGF, but with lower mitogenic capability. They proved in vitro and in vivo that the amount of surface EGFR dictated drug delivery for GE11-EXOs in different breast cancer cell lines. Indeed, the delivery of let-7 miRNA to cancer cells was able to impair cell cycle progression and reduced cell division. Similar inhibitory effects were reported for EXO-mir targeting HER2 [120]. In this case, the authors used mesenchymal stem cell-derived EXOs as carriers of the oncosuppressor miR-145 in T-47D cells, confirming the tumour-suppressive effects of miR-145. In particular, miR-MSC-EXO demonstrated its role in both inducing apoptosis and inhibiting metastasis by downregulating MMP9 and HER2 and upregulating TP53. A different miRNA was tested by Wang et al. with EXOs transporting anti-miR-214. In this case, a link with chemoresistance was evidenced in gastric cancer cells in vitro, showing that EXOs, as vehicles of anti-miR-214, enhanced cell death and inhibited migration in cisplatin-resistant cells [121]. In 2017, EXOs transporting siRNA targeting the oncogenic mutated GTPase KRAS^G12D^ were used to treat pancreatic cancer, yielding suppression of tumour growth and extended survival in mice [122]. In 2019, Limoni et al. obtained promising results in vitro with EXOs containing *TPD52* siRNA in HER2-expressing SKBR3 breast cancer cells [123].

## 6. Conclusions

Although there are several challenges associated with analysing EVs, such as the numerous sources of secretion into body fluids, the lack of standardized isolation and purification protocols, and the absence of a universally agreed-upon marker signature to identify the origin of EVs, their potential use is still significant and promising. An update in the field of Minimal Information for Studies of Extracellular Vesicles has been provided recently by the International Society for Extracellular Vesicles (ISEV). However, more extensive studies are needed to establish a low cost and accurate method for future clinical applications [182,183,184].

EGFR-dependent tumours through the EV cargo of specific proteins, mRNAs, or miRNAs can modulate long-distance and organ-specific cells by promoting growth and invasion, regulating TME, and conferring drug resistance.

Notably, recent studies demonstrated the transfer of EGFR and associated molecules from different cancer cells, activating pro-tumorigenic pathways in recipient cells. The process of transferring biological signals between cells by EV is similar to the hormonal communication and opens new perspectives in the evaluation of the biological effects of such transfer. Whether and how tumour cells sort and transfer cargo in EVs is still an unanswered question. On the other hand, EVs present in liquid biopsies, such as serum, plasma, CSF, and pleural effusions, offer a crucial and untapped opportunity for the development of non-invasive biomarkers that can revolutionize the diagnosis, prognosis, and therapy monitoring not just for EGFR-dependent tumours, but also for a wider range of cancers and diseases.

## Figures and Tables

**Table 1:** Some of the most commonly found proteins in MVs and EXOs [3,26,33,52,65,95].

Biological Role	MVs	EXOs
Membrane Organizers—Adhesion Molecules—Membrane Receptor	Tetraspanins: CD9, CD81, CD82	Tetraspanins: CD9, CD81, CD63, TSPAN6, TSPAN8, CD151, CD37, CD53,
	Integrins, selectins	Flotilin 1 and 2, A33, EpCAM, CD147, Integrin α and β, P-selectin
Biogenesis and Sorting	ARF6, RAB11, ALIX, TSG101, ERK, PLD, VPS4, ESCRT-I, -III, LGALS4	Protein kinases, β-catenin, 14-3-3, G proteins, ALIX, TSG101, syntenin, ubiquitin, clathrin, VPS32, VPS4, ESCRT-0-III, LGALS3BP
Cytoskeletal Proteins	Actin, tubulin, moesin	Tubulin, moesin
Cell Type Specific	MHC-I, CD14, HSP70-90	MHC-I-II, TFR, WNT, CD86, HSP20-27-60-70-84-90
Cancer Associated and Growth Factors	c-Met, Caveolin-1, EGFR, EpCAM, EMMPRIN, MUC1, FAK, HepPar1	TNF-α, TGF-β, TRAIL, GPC1

A33, cell surface A33 antigen; ALIX, programmed cell death-6 interacting protein; ARF6, ADP-ribosylation factor 6; CD, cluster of differentiation; c-Met, hepatocyte growth factor receptor; EGFR, epidermal growth factor receptor; EMMPRIN, extracellular matrix metalloproteinase inducer; EpCAM, epithelial cell adhesion molecule; ERK, extracellular signal-regulated kinase; ESCRT, endosomal sorting complex required for transport; FAK, focal adhesion kinase; HSP, heat shock protein; LGALS, lectin galactoside-binding soluble; MHC, major histocompatibility complex; MUC1, Mucin short variant S1; PLD, phospholipase-D; RAB, Ras-related protein; TfR, transferrin receptor; TGF, transforming growth factor; TNF, tumour necrosis factor; TRAIL, TNF-related apoptosis-inducing ligand; TSG101, tumour susceptibility gene 101; TSPAN, tetraspanin; VPS4, vacuolar protein sorting.
